# Functional Characterization of a Cucumber (*Cucumis sativus* L.) Vacuolar Invertase, CsVI1, Involved in Hexose Accumulation and Response to Low Temperature Stress

**DOI:** 10.3390/ijms22179365

**Published:** 2021-08-29

**Authors:** Zili Feng, Fenghua Zheng, Silin Wu, Rui Li, Yue Li, Jiaxin Zhong, Hongbo Zhao

**Affiliations:** 1 School of Biological Science and Engineering, Shaanxi University of Technology, Hanzhong 732001, China; fengzili@snut.edu.cn; 2 College of Horticulture, South China Agricultural University, Guangzhou 510642, China; 20192017013@stu.scau.edu.cn (F.Z.); wusilin@stu.scau.edu.cn (S.W.); 20193137063@stu.scau.edu.cn (R.L.); 930474894@stu.scau.edu.cn (Y.L.); 3 Centre for Organismal Studies Heidelberg, Department of Plant Molecular Physiology, University of Heidelberg, 69120 Heidelberg, Germany; jiaxin.zhong@cos.uni-heidelberg.de

**Keywords:** *Cucumis sativus* L., invertase, low temperature, hexose, invertase inhibitor

## Abstract

Cucumber (*Cucumis sativus* L.), an important vegetable plant species, is susceptible to low temperature stress especially during the seedling stage. Vacuolar invertase (VI) plays important roles in plant responses to abiotic stress. However, the molecular and biochemical mechanisms of VI function in cucumber, have not yet been completely understood and VI responses to low temperature stress and it functions in cold tolerance in cucumber seedlings are also in need of exploration. The present study found that hexose accumulation in the roots of cucumber seedlings under low temperature stress is closely related to the observed enhancement of invertase activity. Our genome-wide search for the vacuolar invertase (VI) genes in cucumber identified the candidate VI-encoding gene *CsVI1*. Expression profiling of *CsVI1* showed that it was mainly expressed in the young roots of cucumber seedlings. In addition, transcriptional analysis indicated that *CsVI1* expression could respond to low temperature stress. Recombinant CsVI1 proteins purified from *Pichia pastoris* and *Nicotiana benthamiana* leaves could hydrolyze sucrose into hexoses. Further, overexpression of *CsVI1* in cucumber plants could increase their hexose contents and improve their low temperature tolerance. Lastly, a putative cucumber invertase inhibitor was found could form a complex with CsVI1. In summary, these results confirmed that CsVI1 functions as an acid invertase involved in hexose accumulation and responds to low temperature stress in cucumber seedlings.

## 1. Introduction

Sucrose, an important photosynthetic product in plants [1,2,3,4], is translocated from photosynthetic (source) to non-photosynthetic (sink) tissues [3,4,5], and is hydrolyzed into hexoses or their derivates for plant cell growth and development [3,4,5,6,7]. Sucrose synthase (SUS) and acid invertase (INV) participate in this sucrose hydrolysis in higher plants [8]. SUS hydrolyzes sucrose into uridine diphosphate glucose and fructose, reversibly, while INV irreversibly breaks sucrose down into glucose and fructose [9,10]. Depending on their different subcellular locations, INVs are classified into two types: cell wall invertase (CWI) and vacuolar invertase (VI) [11,12,13].

VI plays crucial roles in cell expansion, sugar accumulation and concentration [14,15,16], biomass production [17,18], stomatal conductance [19], and also floral organ development [20]. Besides, VI-derived hexose signals could activate the growth of cotton (upland cotton) fiber (seed trichome) by regulating the expression of certain MYB transcription factors and auxin signaling genes [21].

Apart from the above functions, the genetic manipulation of VIs has provided some surprising results in many plants for enhanced resistance to abiotic stresses [22,23,24] such as hypoxia and gravitropism [19], drought [25,26], and cold [27,28]. One report confirmed that silencing the potato *VI* gene significantly reduced cold-induced sweetening in stored potato tubers [29]. In addition, enhanced VI activity in potato leaf guard cells caused higher stomatal conductance and CO_2_ fixation but decreased water-use efficiency [30]. All of these studies have revealed that under abiotic stress, VI could improve the stress resistance or tolerance of plants via regulating the distribution of carbohydrates and the ratio of hexose to sucrose [19,25,26,27,28,29,30].

Low-temperature stress affects plant growth and development and severely limits both the geographic distribution of plants and crop yields [31]. Cucumber is susceptible to low temperature stress throughout its growth but is particularly so at the seedling stage as the shallow cucumber root system is easily injured by low temperature [32,33,34].

Although VI might be involved in plant low temperature tolerance, no reports regarding this process were found for cucumber. To our knowledge, no acid invertase gene has previously been cloned and functionally identified in cucumber, and mechanisms related to VI-mediated low temperature tolerance have also remained unclear. In the present study, a gene encoding one previously uncharacterized VI-related enzyme, CsVI1, was identified and functionally characterized as involved in sucrose metabolism and responses to low temperature stress in cucumber seedlings.

## 2. Results

### 2.1. Low Temperature Affects Invertase in Cucumis sativus Seedling Roots

**Figure 1:**
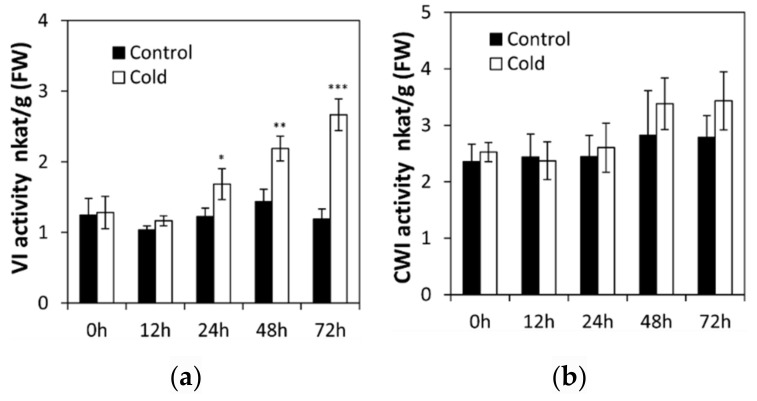
Effect of low temperature treatment of cucumber seedlings on the root vacuolar invertase (**a**) and cell wall invertase (**b**) activities. Cell wall invertase, CWI; vacuolar invertase, VI. Results are means of >3 biological replicates (± SE) each with three technical replicates. Asterisks indicate statistically significant differences (*, *p* value < 0.05; **, *p* value < 0.01; ***, *p* value < 0.001).

To analyze the effects of low temperature stress on seedling, invertase activities were assayed in the roots of cucumber seedlings at the two-leaf stage. Under normal conditions, activities of vacuolar invertase and cell wall invertase did not change significantly from 0 to 72 h (Figure 1a,b). Further, low temperature did not affect cell wall invertase activities (Figure 1b). In contrast, after 24 h of low temperature stress treatment, invertase activities became induced more significantly over time in cucumber seedling roots (Figure 1a).

**Figure 2:**
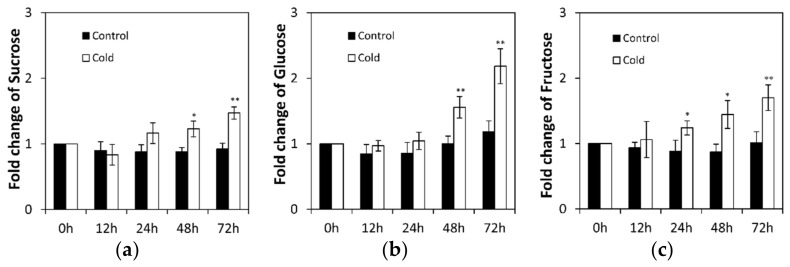
Sucrose (**a**), glucose (**b**), and fructose (**c**) contents in the soluble cell fractions of low temperature-treated cucumber seedling roots. Results are means of >3 biological replicates (± SE) each with three technical replicates. Asterisks indicate statistically significant differences (*, *p* value < 0.05; **, *p* value < 0.01).

To uncover whether low temperature induced invertase activities affect sucrose metabolism, sucrose and hexose (glucose and fructose) contents were measured in cucumber seedling roots under low temperature stress and control conditions (Figure 2). Under normal conditions, sucrose and hexose contents fluctuated to a lesser extent (Figure 2). However, low temperature stress induced a marked sucrose accumulation from 48 to 72 h (Figure 2a). Simultaneously, hexose accumulation increased remarkably (Figure 2b,c). These results indicate that sucrose and hexose accumulation are well correlated to the invertase activity.

### 2.2. Genome-Wide Search for Genes Encoding Vacuolar Invertase in Cucumis sativus

**Figure 3:**
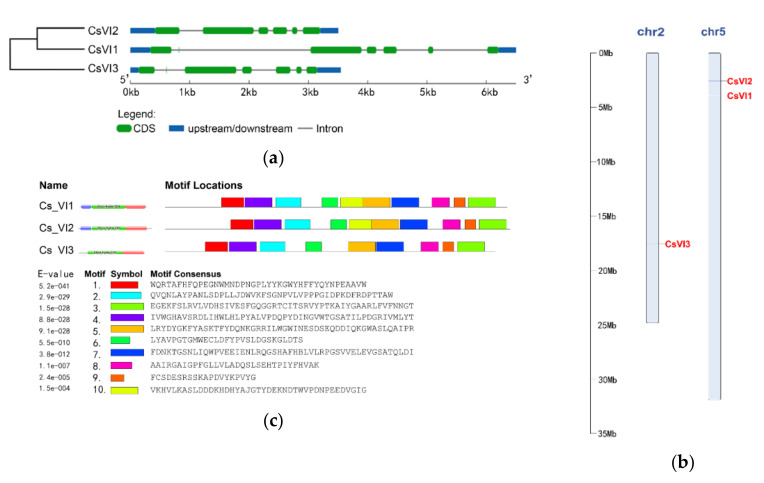
Gene structure (**a**), chromosomal localization (**b**), and conserved protein motif (**c**) of three putative *CsVI* genes.

Previously, no invertase-encoding gene had been characterized from cucumber. Thus, a genome-wide search for genes encoding invertase, especially vacuolar invertase, in cucumber was undertaken in order to understand which invertase gene could be involved in cucumber seedling responses to low temperature. Therefore, a systematic blast of the cucumber genome was performed using the reported VI-encoding genes from *Arabidopsis* and maize as queries. After removal of redundant sequences, three candidate VI-encoding genes were chosen and named *CsVI1* (Csa5G160230.1), *CsVI2* (Csa5G174590.1), and *CsVI3* (Csa2G351670.1). Analyses and comparison of their exon/intron gene structures using GSDS showed that each of these genes contain 6 exons and 5 introns (Figure 3a). Analyses of their chromosomal localizations indicated that *CsVI1* and *CsVI2* are located on chromosome 5, while *CsVI3* is located on chromosome 2 (Figure 3b). Conserved motifs in the CsVI protein sequences were identified using Pfam (32.0) and MEME. Nine conserved motifs were detected in all three CsVIs by MEME, and one other conserved motif was found only in CsVI1 and CsVI2 (Figure 3c).

To predict any possible regulation of gene expression by environmental or phytohormone stimuli, the presence of *cis*-regulatory elements in the promoter of the *CsVI*s gene was analyzed using the PlantCARE database. Four *cis*-regulatory elements putatively involved in regulation of gene expression by phytohormones, seven related to induction of gene expression by abiotic stress, and one related to regulation of zein metabolism were detected (Figure S1). A MYB-binding site, a MeJA response motif, and an anaerobic induction motif were found in the promotors of all three of these *CsVI* genes. These results allowed us to form hypotheses about stress-regulated expression of *CsVI* genes.

### 2.3. Expression Profiling of CsVIs in Cucumber Seedlings

**Figure 4:**
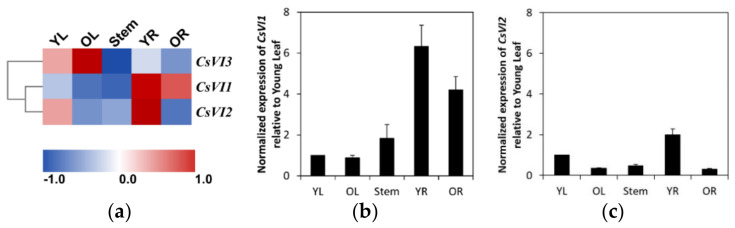
Expression profiles of *CsVI* genes in different cucumber tissues. (**a**) Transcriptomic analysis of the expression of *CsVI*s illustrated as a heat map. The scale represents the range of gene expression of *CsVI*s as RPKM in different samples after *Z*-Score standardization [35]. (**b**) Real-time PCR analysis of transcript abundance of *CsVI1*. (**c**) Real-time PCR analysis of the transcript abundances of *CsVI2*. Young leaf, YL; older leaf, OL; Young root, YR; older root, OR. Results are means of >3 biological replicates (± SE) each with three technical replicates.

To explore whether cucumber *CsVI*s are expressed in a tissue-specific manner, transcriptomic data for different cucumber seedling tissues were analyzed and the results are illustrated as a heat map (Figure 4). *CsVI1* is mainly expressed in roots, especially in young cucumber roots (Figure 4a); *CsVI2* is mainly expressed in young roots and young leaves (Figure 4a); and *CsVI3* is mainly expressed in leaves, especially in older leaves (Figure 4a). Additional analyses of the expression of these genes by real-time PCR revealed that *CsVI1* expression could be detected in roots, stems, and leaves of cucumber seedlings, and that high *CsVI1* expression predominate in young roots (Figure 4b). *CsVI2* expression could be detected in the entire seedlings, but its expression is particularly stronger in young tissues like young leaves and young roots (Figure 4c). From these results, we can hypothesize that the expression of *CsVI1* and *CsVI2* might help young cucumber roots adapt to a variable environment by modulating their growth. Further, real-time PCR indicated almost no expression of *CsVI3* in young cucumber seedling tissues (data not shown), which suggests that this gene may exhibit other spatial and temporal expressions during cucumber growth and development.

### 2.4. CsVI1 Transcript Abundance Responds to Low Temperature Stress but Not Heat Stress in Cucumber Seedling Roots

**Figure 5:**
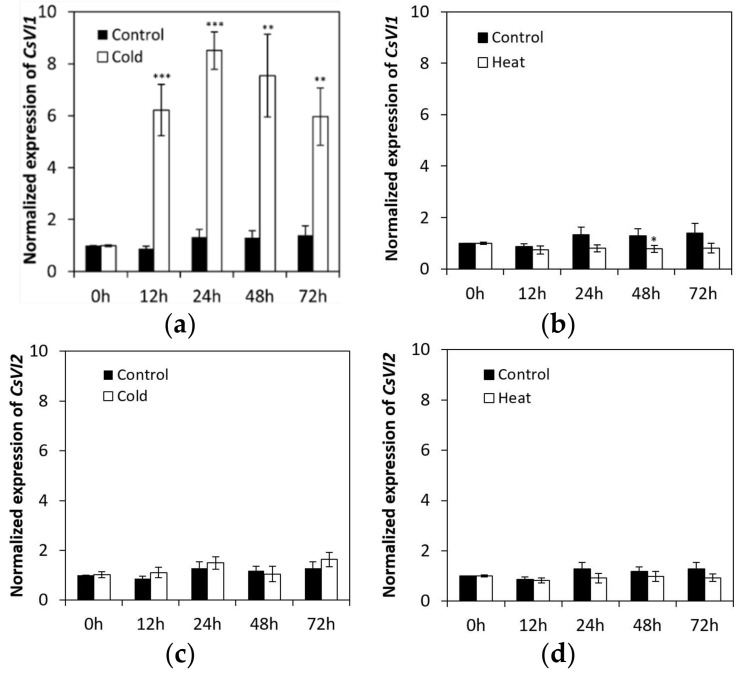
Effects of low temperature or heat treatments on the expression of *CsVI1* (**a**,**b**) and *CsVI2* (**c**,**d**) in cucumber seedling roots. Results are means of >3 biological replicates (± SE) each with three technical replicates. Asterisks indicate statistically significant differences (*, *p*-value < 0.05; **, *p*-value < 0.01; ***, *p*-value < 0.001).

Under low temperature treatment, the abundance of *CsVI1* transcripts increased significantly (Figure 5a), but under heat treatment the abundance of *CsVI1* transcripts decreased slightly (Figure 5b). In accordance with the results for *CsVI1* expression, cold treatment also specifically enhanced the activity of INV (Figure 1a) but not that of CWI (Figure 1b). Neither low temperature nor heat treatments markedly modulated the transcript expression of *CsVI2* (Figure 5c,d). These results indicate that *CsVI1* transcript abundance was specifically related to the low temperature response in cucumber seedling roots. In contrast, no expression of *CsVI3* transcripts could be detected by cold and heat treatments (data not shown).

### 2.5. Characterization of the CsVI1 Protein and Analysis of Invertase Activity

**Figure 6:**
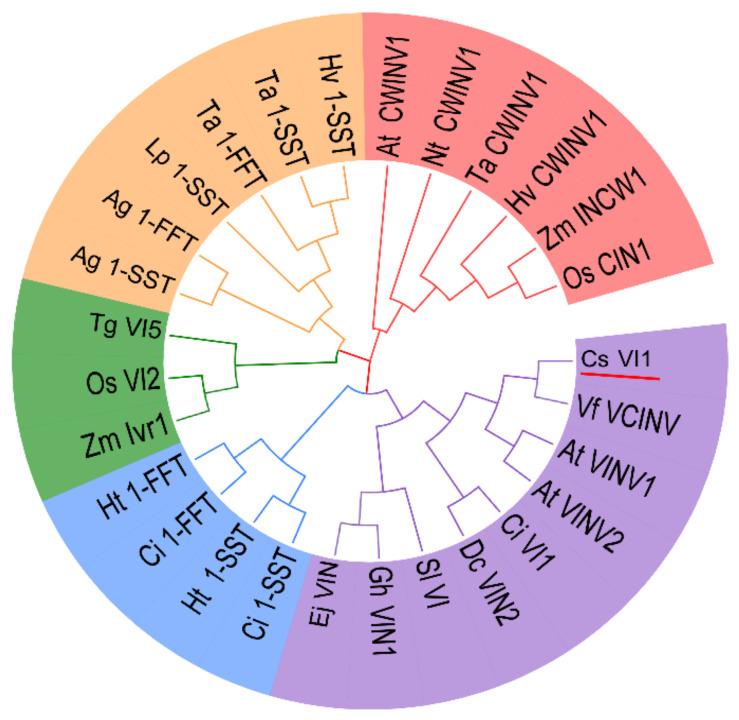
Phylogenetic analysis of cDNA-derived amino acid sequences of vacuolar invertase and vacuolar invertase-like predicted proteins from cucumber and other plant species. Five groups can be distinguished. Groups shown in green and purple are classified as vacuolar invertase (VIs). The VIs from monocot species are shown in green, while those from dicot species are shown in purple. The cell wall invertase group is shown in red. Groups shown in yellow and blue are classified as fructan biosynthetic enzymes (FBEs). CsVI1 is underlined. Plant species are abbreviated as follows: At, *Arabidopsis thaliana*; Ag, *Agave tequilana*; Ci, *Cichorium intybus*; Cs, *Cucumis sativus*; Dc, *Daucus carota*; Ej, *Eriobotrya japonica*; Gh, *Gossypium hirsutum*; Ht, *Helianthus tuberosus*; Nt, *Nicotiana tabacum*; Hv, *Hordeum vulgare*; Lp, *Lolium perenne*; Os, *Oryza sativa*; Sl, *Solanum lycopersicum*; Ta, *Triticum aestivum*; Tg, *Tulipa gesneriana*; Vf, *Vicia faba*; and Zm, *Zea mays.* Phylogenetic and molecular evolutionary analyses were conducted using MEGA7 (https://www.megasoftware.net/, accessed on 7 January 2019) by neighbor-joining analysis [39].

**Figure 7:**
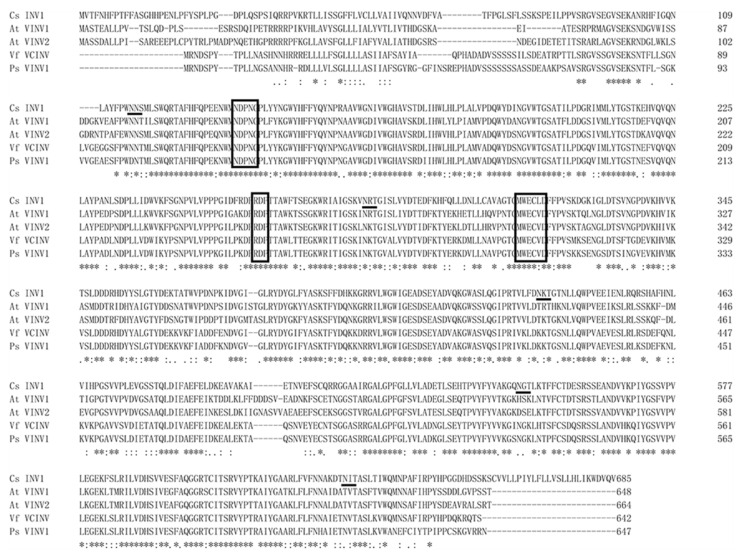
Alignment of the CsVI1 amino acid sequence with vacuolar invertases from other dicot species. The amino acid sequences were aligned using ClustalW Omega (https://www.ebi.ac.uk/Tools/msa/clustalo/, accessed on 11 March 2019). Putative β-fructosidase motifs (NDPNG/A), cysteine-containing catalytic sites (MWECP/V), and conserved Asp residues (D) are shown in boxes. Putative glycosylation sites are underlined. Asterisks indicate identical residues, colons indicate conserved substitutions, and periods indicate semi-conserved substitutions.

To confirm whether *CsVI1* encodes an actual invertase, the full-length protein sequence of CsVI1 was compared with the amino acid sequences of other functionally identified INVs. As Figure 6 shows, in the evolution, CsVI1 was phylogenetically close to VI from grape (VfVCINV) and two VIs from *Arabidopsis* (AtVINV1 and AtVINV2). As previous studies have indicated that FBEs (fructan biosynthetic enzymes) could have evolved from VIs [36,37,38], further phylogenetic analyses showed that CsVI1 was not grouped together with the FBEs but was instead grouped very closely to the VIs (Figure 6). Additionally, the predicted sequence of CsVI1 showed three conserved invertase motifs, the β-fructosidase motif NDPNG/A, the cysteine-containing catalytic site MWECP, and the conserved Asp residues (D) (Figure 7) characteristic of VIs. Further, as shown for invertases from other plant species, mature invertase protein are post-translationally glycosylated [11,38]. As expected, five predicted glycosylation sites were identified in the predicted CsVI1 protein (Figure 7).

**Figure 8:**
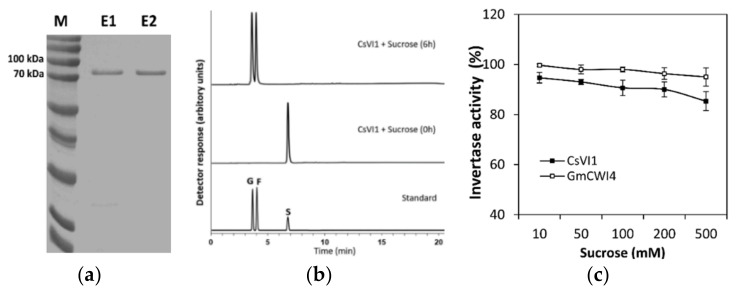
Detection of CsVI1 enzyme activity. (**a**) SDS-PAGE analysis of recombinant CsVI1 protein from *Pichia pastoris*. Marker (M), elution fractions (E1–2) in 100 mM imidazole. (**b**) Analysis of INV enzyme activity of recombinant CsVI1 (20 μg) with sucrose (50 mM) as a substrate. (**c**) Comparison of INV enzyme activities between recombinant CsVI1 and the previously identified acid invertase GmCWI4. Sucrose (10–500 mM) was incubated with the recombinant proteins (20 μg). The fructose and glucose liberated from sucrose were measured using HPAEC-PAD. The standards used for HPAEC-PAD included: G, glucose; F, fructose; and S, sucrose. The term “invertase activity %” means percentage of sucrose hydrolyzed. Results are means of >3 biological replicates (± SE) each with three technical replicates.

**Figure 9:**
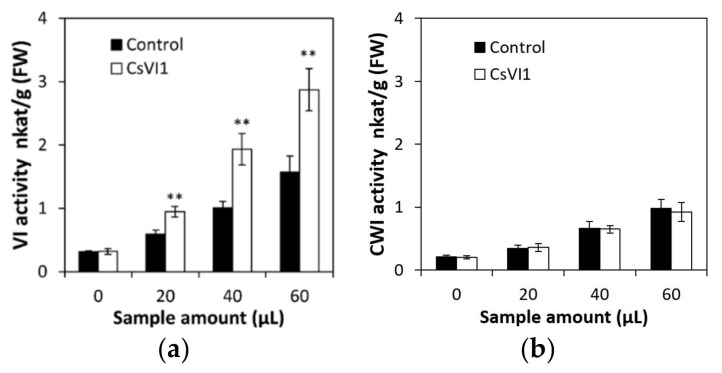
Cell wall and vacuolar invertase activities in *Nicotiana benthamiana* leaves transiently transformed with *CsVI1* (**a**,**b**). A substrate concentration of 100 mM sucrose was used to assay cell wall and vacuolar invertase activities. The invertase activity induced by transformation with the empty vector alone was subtracted. Results are means of >3 biological replicates (± SE) each with three technical replicates. Asterisks indicate statistically significant differences (**, *p*-value <0.01).

To clarify whether the CsVI1 enzyme can use sucrose as a substrate, the INV activity of a purified recombinant CsVI1 protein from a *Pichia pastoris* heterological expression system (Figure 8a) was tested and the fructose and glucose liberated from hydrolyzed sucrose was quantified by HPAEC-PAD. Results showed that sucrose was degraded into hexose (glucose and fructose) in the presence of recombinant CsVI1 protein after 6 h (Figure 8b). GmCWI4, a previously identified invertase from *Glycine max* [40], was used for comparison of enzymatic activity to CsVI1. Results showed that like GmCWI4, the purified recombinant CsVI1 protein showed similar sucrose hydrolyzation activity with increasing sucrose concentration (Figure 8c), demonstrating the invertase activity CsVI1 and that its enzymatic activity was not affected by any variation in sucrose concentration. Furthermore, to determine whether CsVI1 exhibits invertase activity in vacuolar in vivo, the *Nicotiana benthamiana* leaves system was used to transiently express CsVI1 protein. As expected, the results showed that CsVI1 significantly elevated the invertase activity in the vacuole but not in the cell wall (Figure 9).

### 2.6. Overexpression of CsVI1 Increases the Hexose Content and Low Temperature Tolerance of Cucumber Seedling Roots

**Figure 10:**
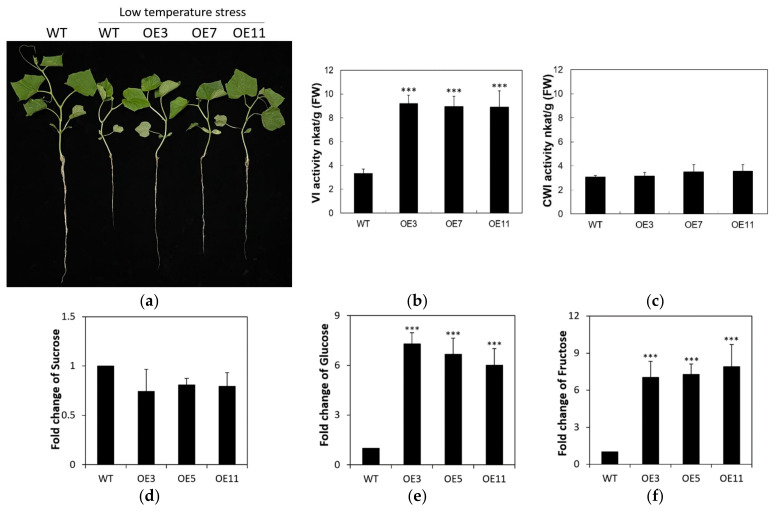
*CsVI1* overexpression lines and WT after 72 h low temperature treatment. (**a**) Phenotype of WT and *CsVI1* overexpression lines. (**b**) Vacuolar invertase activities and (**c**) cell wall invertase activities in root tissues of WT and *CsVI1* overexpression lines. (**d**) Variations in sucrose, (**e**) glucose and (**f**) fructose contents in root tissues of WT and *CsVI1* overexpression lines. Results are means of >3 biological replicates (± SE) each with three technical replicates. Asterisks indicate statistically significant differences (***, *p*-value <0.001).

To analyze the role of CsVI1 in cucumber during low temperature stress, three independent transgenic lines carrying the *CsVI1* overexpression construct were developed. Phenotypic analyses showed that the *CsVI1* overexpression lines exhibited enhanced low temperature tolerance in cucumber seedlings, especially as reflected in improved root growth (Figure 10a), increased vacuolar invertase activities (Figure 10b), and higher hexose contents (Figure 10e,f) as compared with the wild type seedling roots. However, no significant changes in cell wall invertase activity (Figure 10c) or sucrose content (Figure 10d) were found in cucumber seedling roots of *CsVI1* overexpression lines.

### 2.7. A Putative Cucumber Invertase Inhibitor CsINVINH1 Can Form a Complex with CsVI1

Invertase inhibitors can post-translationally regulate invertase activity [41]. So far, no studies of invertase inhibitor function in cucumber have been published. To reveal possible interactions between CsVI1 and a hypothesized cucumber invertase inhibitor, a putative cucumber invertase inhibitor-encoding sequence, named *CsINVINH1* (Cucsa.302150), was screened in the cucumber genome and used for protein–protein interaction modeling described below.

**Figure 11:**
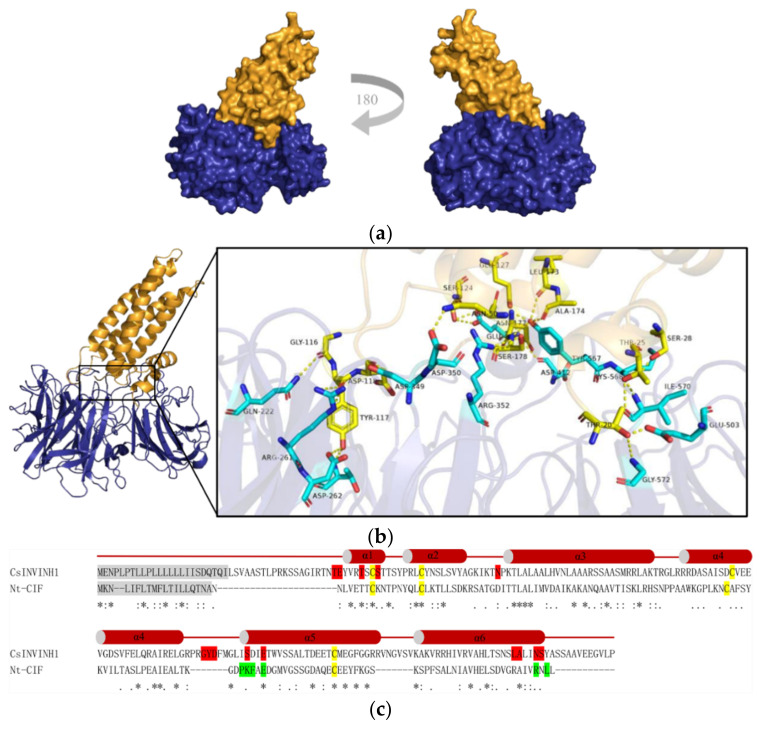
Modeled structure of proposed CsVI1/CsINVINH1 complex shows that CsINVINH1 might interact with CsVI1. (**a**) Overall structure of the proposed CsVI1 (blue)/CsINVINH1 (yellow) complex. (**b**) The interface between the proteins is comprised of conserved amino acids or motifs of the modeled interacting proteins (right, close-up view of the proposed interacting regions). The yellow amino acids are from CsINVINH1, and the blue amino acids are from CsVI1. Hydrogen bonds are shown as yellow dotted lines. (**c**) Amino acid sequence comparison of CsINVINH1 and NtCIF. For the proposed CsVI1/CsINVINH1 complex, all the putative binding sites in CsINVINH1 are shaded in red. For the AtCWI1/NtCIF complex, the key binding sites in NtCIF are shaded in green. The signal peptide sequence is shaded in gray, and an invariant cysteine of invertase inhibitors in yellow. Asterisks indicate identical residues, colons indicate conserved substitutions, and periods indicate semi-conserved sub-stitutions.

The possible interaction between CsVI1 and CsINVINH1 was modeled using the complex structure of AtCWI1 and the tobacco invertase inhibitor NtCIF [42] as template. We predicted the interaction of CsINVINH1 with the active site of CsVI1 via its sequence motif GLISDIE that corresponds to the GDPKFAE motif in NtCIF (Figure 11 and Figure S2). In helix α5 of CsINVINH1, two amino acids (Ser124 and Glu127) might directly contact the Glu414 residue of CsVI1 (Figure 11b,c and Figure S2). Ser124 of CsINVINH1 corresponds to Lys98 of NtCIF, so in principle, it might interact with a fructose molecule or Glu414 of CsVI1. In helix α6 of CsINVINH1, four amino acids (Ser178, Asn177, Leu173, and Ala174) might bind the Try567 residue of CsVI1 (Figure 11b,c and Figure S2). The important amino acids Asp349/Arg352 in CsVI1 (comparable to Asp239/Lys242 in AtCWI1) might hold the glucose moiety of sucrose in a catalytically competent position, illustrated by the proposed binding of Asp349 to Asp118 of CsINVINH1 and the proposed interaction of Arg352 with Ser178 of CsINVINH1 (Figure 11b,c and Figure S2). Further, Thr20, Thr25, Ser28, Gly116, Try117, and Asn177 in CsINVINH1 might bind to Gly572 and Glu503; Ile570; Lys568; Gln222; Thr198 and Asp262, and Arg261; and Asp412 in CsVI1, respectively Figure 11b,c and Figure S2).

## 3. Discussion

Most plants adapted to temperate regions respond to chilling low temperatures (2 to 6 °C) through processes including gene expression changes, metabolite reprogramming, and cellular and tissue structural remodeling. Cucumber is a vegetable species that is particularly sensitive to chilling. In the present study, we found that VI activity increased in cucumber seedling roots exposed to low temperature conditions and that the sucrose hydrolysis activity also increased. We suggest that VI might function as a regulator of osmotic potential in cucumber seedling cells by cleaving single sucrose molecules into two hexose (fructose and glucose) molecules in the vacuoles. That is, the high VI activity induced by low temperatures results in higher glucose and fructose contents, which might induce a potential osmotic change that could help cucumber seedling cells tolerate low temperature stress. Indeed, overexpression of *CsVI1* did promote an increase in monosaccharide content (Figure 10e,f), which could help to maintain a favorable osmotic potential, sustain normal water inflow required by the cells, protect the integrity of the plasma membrane from water loss, and finally prevent or decrease the damage caused by low temperature. In one study that supports this assumption, cold acclimation in tea plants was found to induce increases in glucose and fructose contents, increase the hexose to sucrose ratio, and enhance tea plant tolerance to cold stress [27]. Further analysis of the expression of genes related to osmotic adjustment (*P5CS1, P5CS2, AtLEA3, COR413-PM1,* and *COR15B*) indicated that hexoses might be related to the changes in osmotic potential observed in tea plants in that study [27]. Further, previous work in *Arabidopsis*, has shown that VI can adjust osmotic potential in cells by increasing sugar content [43].

The shallow root system of cucumber is easily injured by low temperature [32,33,34], however, low temperature induction of *CsVI1* expression and function might be able to alleviate the negative effects of cold stress on cucumber seedling root growth. High-density long lateral roots are promising features for improving the early vigor of seedlings under unsatisfactory soil temperature conditions [44]. To overcome the impacts of low temperature stress, the induced expression of *CsVI1* might provide a suitable root cap ratio by increasing both the root elongation rate and biomass (Figure 10a), thereby making them better able to absorb the nutrients and water. Some studies have found that VI can promote plant growth processes such as cell expansion and cell division [21,27,45]. For example, overexpression of the tea vacuolar invertase gene *CsVI5* in *Arabidopsis* roots enhanced both taproot and lateral root elongation [27]. Further, the cotton vacuolar invertase1 *GhVIN1* was found to be a major player in regulating cotton fiber cell elongation [21,46]. These studies, together with ours, implicate that VI can improve plant growth, especially root growth, may be via regulating the distribution of carbohydrates and increasing the ratio of hexose to sucrose.

Hexoses are thought to act as signaling molecules during plant growth and development [47], and hexose formation might affect sugar sensing networks [48]. In plants, a hexose kinase has been found to engage in cross-talk with signaling by phytohormones such as ABA, ethylene, and MeJA [1,49,50,51], which might help plants to resist low-temperature stress. We propose that *CsVI1* participates in the low-temperature tolerance in cucumber seedling roots by increasing hexose concentrations, which subsequently triggers downstream signal transduction, perhaps phytohormone metabolism.

Under abiotic stresses, the concentrations of reactive oxygen species (ROS) increased dramatically, which significantly damaged cell structures, and some studies have found that sucrose and hexoses are involved in ROS scavenging [52,53]. Previous work in cucumber chloroplast has shown that chilling-insensitive genotypes have higher ROS scavenging capacity to resist oxidative stress [54]. Whether CsVI1 can decrease ROS concentrations in cucumber seedlings to alleviate the symptom of low temperature stress remains to be clarified. Furthermore, whether induction of sucrose hydrolysis in the vacuole can retard or block cytosolic sucrose metabolism and enhance cucumber seedling tolerance to low temperature also needs further study.

VI activity can be post-translationally regulated by an invertase inhibitor [41]. Our modeling of the proposed interaction between CsVI1 and CsINVINH1 by comparison with the crystal structure of the AtCWI1-NtCIF-complex [42] demonstrated that a similar mode of interaction between the cucumber proteins is possible (Figure 11), with conserved amino acids and motifs interacting at the inhibitory enzyme interface. CsINVINH1 could interact with the active site of CsVI1 via its sequence motif GLISDIE, which corresponds to the GDPKFAE motif in NtCIF (Figure 11; Figure S2). Additionally, the amino acids Asp349/Arg352 in CsVI1 might hold the glucose moiety of sucrose in position to bind to Asp118 and Ser178 of CsINVINH1, respectively (Figure 11b,c; Figure S2). Finally, another small amino acid motif from CsINVINH1 might block invertase activity by direct insertion into the substrate binding cleft of CsVI1 (Figure 11b,c; Figure S2). All of these suggest that CsINVINH1 might act as an invertase inhibitor to post-translationally regulate CsVI1 activity in cucumber.

In conclusion, these results confirmed that CsVI1 functions as an acid invertase involved in hexose accumulation and responds to low temperature stress in cucumber seedlings, and CsINVINH1 might post-translationally inhibitor CsVI1 activity.

## 4. Materials and Methods

### 4.1. Plant Material and Treatments

Seedlings of cucumber (*Cucumis sativus* cv. HC no.1) and *Nicotiana benthamiana* L. were cultivated in the greenhouse at 23 ± 2 °C under long day conditions (16 h light period; 300 μmol m^−2^ s^−1^). For cucumber, seedlings at two leaf stage were chosen for 4 °C treatment. In 0 h, 12 h, 24 h, 48 h, and 72 h, tissue samples collected were either used immediately or frozen in liquid nitrogen and stored at −80 °C until use. For *Nicotiana benthamiana*, leaves from 8 to 12 weeks plants were used for transient transformation by leaf infiltration with *Agrobacterium tumefaciens* strain C58C1 cells harboring appropriate plasmids (see Section 4.4). After 48 h, leaf samples were collected for use immediately or frozen in liquid nitrogen and stored at −80 °C until use.

### 4.2. Gene Structure, Distribution, Cis-Element, and Conserver Motif Analysis

The sequences for the candidate invertase genes were obtained from the CuGenDB (http://cucurbitgenomics.org/, accessed on 10 October 2018). Gene structures including the distribution of exons and introns were analyzed using GSDS (http://gsds.cbi.pku.edu.cn/, accessed on 11 March 2019). Alignments of mRNA sequences were performed using MUSCLE (https://www.ebi.ac.uk/Tools/msa/muscle/ accessed on 11 March 2019). Amino acid sequences were aligned using ClustalW Omega (https://www.ebi.ac.uk/Tools/msa/clustalo/, accessed on 11 March 2019). Conserved motifs in proteins were identified using Pfam (http://pfam.xfam.org/, accessed on 11 January 2019) and MEME (http://meme-suite.org/index.html, accessed on 11 March 2019).

The chromosomal distributions of candidate genes were obtained from the (http://cucurbitgenomics.org/, accessed on 10 October 2018) and were illustrated using TBtools (https://www.biorxiv.org/content/10.1101/289660v3, accessed on 3 June 2019). The upstream 1.5-kb sequences of genes were searched for *cis*-regulatory elements using PlantCARE (http://bioinformatics.psb.ugent.be/webtools/plantcare/html/, accessed on 3 June 2019).

### 4.3. Transcriptomic Sequencing and Gene Expression Analysis

Transcriptome sequencing data for different cucumber tissues (BioProject: PRJNA312872) were collected from CuGenDB (http://cucurbitgenomics.org/, accessed on 10 October 2018). TBtools (https://www.biorxiv.org/content/10.1101/289660v3, accessed on 3 June 2019) was used to visualize the expression of the candidate genes. For the quantitative real-time PCR (qPCR) analysis, RNA extraction and cDNA synthesis were performed according to the methods described in a previous report [55]. An additional step was performed with the Rotor-Gene 3000 system using SYBR Premix ExTaq II (Takara, Shiga, Japan) to monitor dsDNA synthesis. The expression level of the target gene was quantified by comparing to the expression of reference gene *tubulin*. Primers for reference gene and target gene are presented in Table S1. For each sample, three independent cDNA preparations were analyzed with three technical replica each.

### 4.4. Plant Transformation

For transient expression of the CsVI1 protein, the coding region of the *CsVI1* gene was cloned into the pB7WG2 vector downstream of the 35S promoter (for primers see Table S1). Transient expression of CsVI1 in *Nicotiana benthamiana* leaves was performed by leaf infiltration with transformed *Agrobacterium* as described in Wolf et al. [56]. Transformation with P19 served as a control to account for induction of any endogenous INV activities due to *Agrobacterium* transformation. Cucumber transformation was carried out using the cotyledon transformation method as previously reported [57]. Positive transgenic plants were verified by PCR and expression of *CsVI1* by the transgenic plants was further assayed by qPCR.

### 4.5. Plant Protein and Soluble Carbohydrate Extraction

The extraction of vacuolar and cell wall-bound proteins from cucumber and *Nicotiana benthamiana* leaves essentially followed the protocol described in Link et al. [58]. Bound proteins were eluted from the resuspended cell wall fraction with 500 mM NaCl (Sangon, Shanghai, China) for 1 h at 4 °C, using an overhead shaker, followed by centrifugation at 10,000× *g* at 4 °C. Soluble and salt-eluted proteins (cell wall-bound fraction) were washed and concentrated by centrifugal filter (Millipore, Darmstadt, Germany) with 50 mM NaOAc (Sangon, Shanghai, China) buffer pH 5. Protein concentrations were determined by Bradford assay (Sangon, Shanghai, China). For total soluble carbohydrates extraction from cucumber seedlings, method as described by Wei et al. [59].

### 4.6. Heterologous Expression and Purification of CsVI1

For the generation of *Pichia pastoris* expression plasmid, restriction sites *Kpn*I and *Apa*I (NEB, Beijing, China) were used to clone the coding region of CsVI1 into the pPICZα vector (Invitrogen, Carlsbad, CA, USA). The ligation product was then transformed into *E. coli* competent DH5α cells by electroporation. Subsequently, the transformed bacterial cells were plated on low-salt LB medium supplemented with zeocin as a selection marker. Positive colonies were then used for vector amplification. The pPICZα plasmid (see above) carrying *CsVI1* (and empty vector as a control) was linearized using *Pme*I, and then transformed into *Pichia pastoris* strain X-33 via electroporation. Further selection and protein purification were performed as previously described [37]. In short, after induction of the expression of CsVI1 recombinant protein in baffled Erlenmeyer flasks, the supernatant was precipitated with 80% ammonium sulfate. The protein pellets were then re-dissolved and dialyzed overnight. His6-tagged recombinant proteins were subsequently purified with 500 mg Ni-IDA (Macherey-Nagel, Allentown, PA, USA) according to the manufacturer’s instructions. Finally, the recombinant protein was eluted from the column with elution buffer.

### 4.7. Enzyme Activity and Carbohydrate Assay

GmCWI4 recombinant protein was kindly gifted by Su et al. [40]. CSVI1 or GmCWI4 protein was incubated with 110–500 mM sucrose (Sangon, Shanghai, China) in 50 mM NaOAc (Sangon, Shanghai, China) buffer, pH 5.0 at 37 °C for different time intervals. After incubation, the reaction was stopped by heating at 95 °C for 5 min. Released fructose and glucose were determined using HPAEC-PAD as described in Wei et al. [60]. In parallel, glucose and fructose were also determined using a coupled spectrophotometric enzyme assay as described in Link et al. [58]. All enzyme measurements were performed under conditions in which activities were proportional to enzyme amounts and incubation times.

### 4.8. Invertase-Invertase Inhibitor Complex Modelling

Homology-based modeling of CsVI1 in a proposed complex with CsINVINH1 was based on the structure of the complex between AtCWI1 and the tobacco invertase inhibitor Nt-CIF [61] (PDB entry 2XQR, https://www.rcsb.org/, accessed on 15 May 2021). Structural alignments and model calculations were done using Modeller 9.25 software [62]. Protein docking analysis was performed using Discovery Studio 2020 (Biovia) and visualized in Discovery Studio Visualizer 2020 (Biovia). High-resolution rendering was performed using PyMOL (https://pymol.org/2, accessed on 20 May 2021) to verify the binding ability of the modeled protein complex.

### 4.9. Statistical Analysis

Statistical analysis was performed using SPSS software (version 17.0, SPSS Institute, Chicago, IL, USA). Each data set was compared with data from the same time point (or the same sample amount) measured under control conditions. ANOVA followed by Student’s t-test was used for mean separation. Different numbers of asterisks represent differences at various levels of significance (i.e., *, *p*-value < 0.05; **, *p*-value < 0.01; ***, *p*-value < 0.001).

## Data Availability

Not applicable.

## Supplementary Materials

The following are available online at https://www.mdpi.com/article/10.3390/ijms22179365/s1.
