# Supplementary material for: Functional Characterization of a Cucumber (Cucumis sativus L.) Vacuolar Invertase, CsVI1, Involved in Hexose Accumulation and Response to Low Temperature Stress
Source: Int J Mol Sci. 2021 Aug 29;22(17):9365. doi: 10.3390/ijms22179365 (PMC8431200; doi:10.3390/ijms22179365)
Supplement: Supplementary file 1 [file ijms-22-09365-s001.zip › ijms-1352561-supplementary.pdf]

Supplementary Materials:

Table S1. Primers used for PCR amplification and cloning. Included are primers for full length constructs (gateway); partial cDNAs for qPCR (qPCR); for heterologous expression of mature proteins (pPICZ).

cDNA	Comment	Primer name	Primer sequence (5′- 3′)
VI1	gateway	VI1GW-F	GGGGACAAGTTTGTACAAAAAAGCAGGCTCCATGGTGA CTTTTAATCATTTTC
		VI1GW-R	GGGGACCACTTTGTACAAGAAAGCTGGGTCTCAAACTT GCACATCCCATTTAAT
	pPICZ	VI1pPICZ-F	ACCGGGGTACCGTCATGGTGACTTTTAATCAT
		VI1pPICZ-R	GACTAGGGGCCCAACTTGCACATCCCATTTAAT
	qPCR	VI1-F	CATTCCCCTTCCCATTCATA
		VI1-R	ATGGCGACCAATAAGCAAAC
tubulin	qPCR	tubulin-F	ACGCTGTTGGTGGTGGTAC
		tubulin-R	GAGAGGGGTAAACAGTGAATC

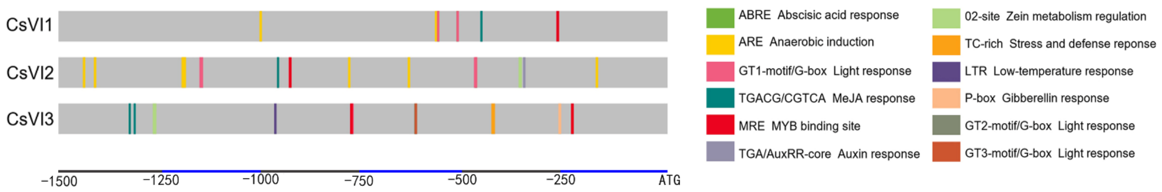

Figure S1. *In silico* analysis of cis-regulatory elements in CsVIs promoters. 1.5 kb sequences from upstream of each gene were used to analysis by PlantCARE (http://bioinformatics.psb.ugent.be/webtools/plantcare/html/). Stress and phytohormone related cis-regulatory elements are boxed with different colors.

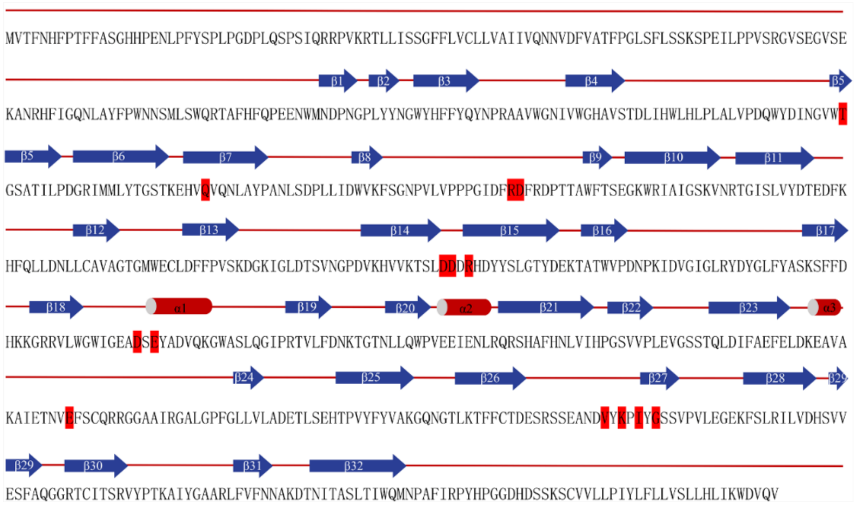

Fig. S2. CsVI1 interacts with CsINVINH1. Binding sites of amino acids in CsVI1 are marked by red. α-helices and β-sheets are marked with columns and arrows, respectively.
